# Supplementary material for: Low input capture Hi-C (liCHi-C) identifies promoter-enhancer interactions at high-resolution
Source: Nat Commun. 2023 Jan 17;14:268. doi: 10.1038/s41467-023-35911-8 (PMC9845235; doi:10.1038/s41467-023-35911-8)
Supplement: Supplementary file 1 — Supplementary Information [file 41467_2023_35911_MOESM1_ESM.pdf]

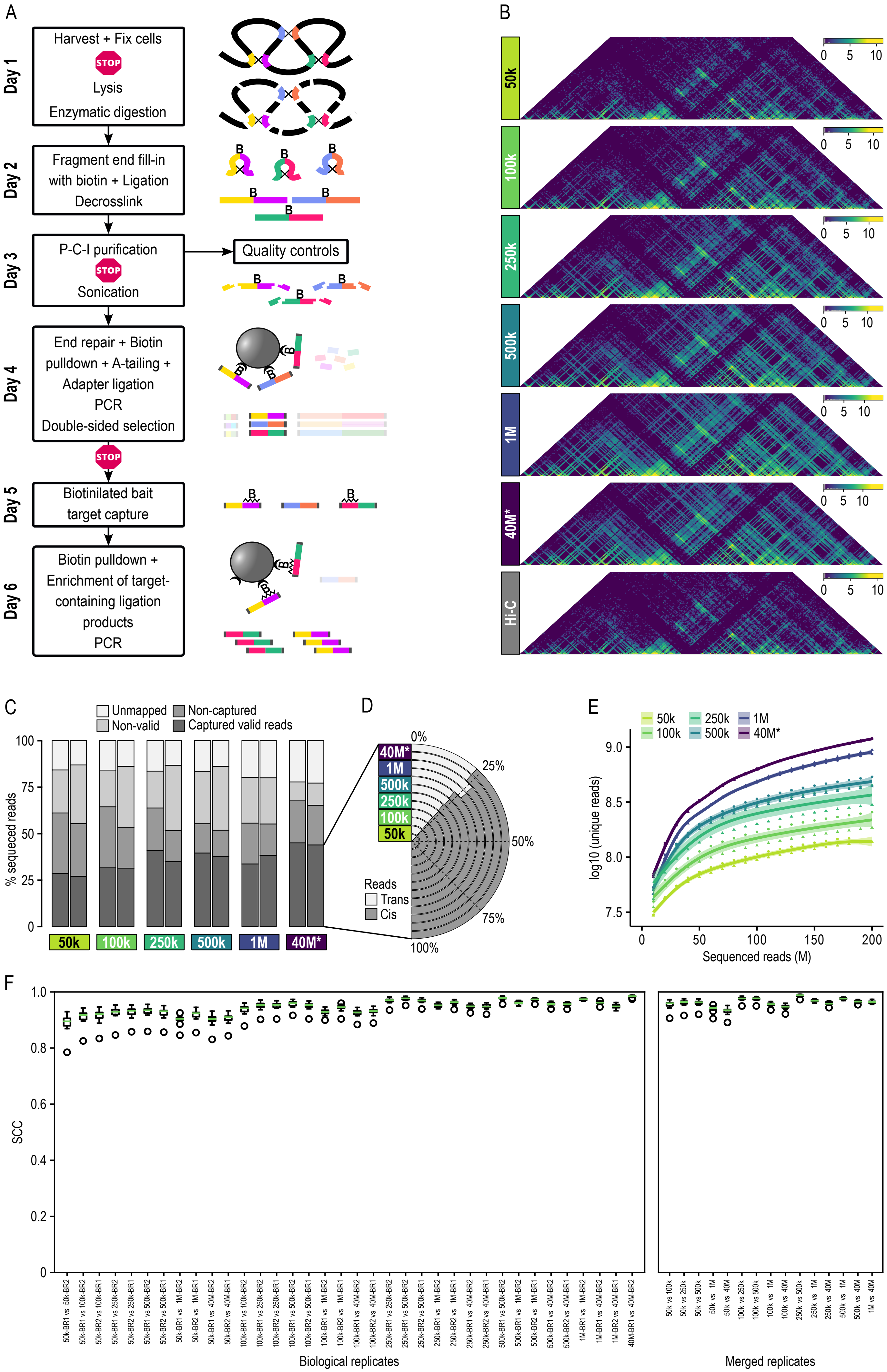
A
B
Day 1
Harvest + Fix cells
STOP
Lysis
Enzymatic digestion
Day 2
Fragment end fill-in with biotin + Ligation
Decrosslink
Day 3
P-C-I purification
STOP
Sonication
Quality controls
Day 4
End repair + Biotin pulldown + A-tailing + Adapter ligation
PCR
Double-sided selection
STOP
Day 5
Biotinilated bait target capture
Day 6
Biotin pulldown + Enrichment of target-containing ligation products
PCR
50k
100k
250k
500k
1M
40M*
Hi-C
0 5 10
C
Unmapped
Non-valid
Non-captured
Captured valid reads
% sequenced reads
100
75
50
25
0
D
0%
25%
50%
75%
100%
Reads
Trans
Cis
E
log10 (unique reads)
9.0
8.5
8.0
7.5
Sequenced reads (M)
0
50
100
150
200
F
SCC
1.0
0.8
0.6
0.4
0.2
0
Biological replicates
Merged replicates

Supplementary Fig. 1 | A In-depth overview of the liCHi-C workflow. “STOP” icons symbolize points in which the protocol may be safely stopped for up to several weeks by freezing the material. Optional quality controls of the first part of the protocol before sonicating may be performed at the expense of an extra day. **B** Interaction matrices of liCHi-C, PCHi-C (*) and Hi-C binned at 50kb resolution of experiments with different numbers of input cells. 1000 (k), million (M). **C** Proportions of reads passed through the different steps of HiCUP. **D** Cis-trans interaction ratio of valid captured reads. Cis and trans mean interactions within and between chromosome respectively. **E** Saturation plot representing unique reads respect to total number of sequenced reads. **F** Boxplots of the stratum adjusted correlation coefficient (SCC) between promoter interactomes of human naive B cells obtained by PCHi-C (*) and liCHi-C using different cell numbers at 100 kb resolution. Medians are represented by green lines. Each boxplot corresponds to the comparison between biological replicates (left) or merged replicates of experiments of different numbers of input cells (right). n=2 biologically independent libraries examined over 6 independent experiments. Boxes expand the interquartile range (IQR), *i.e.*, from the first quartile (Q1) to the third quartile (Q3) values of the data, where the green lines represent the median. Whiskers correspond to the highest and the lowest points with 1.5xIQR.

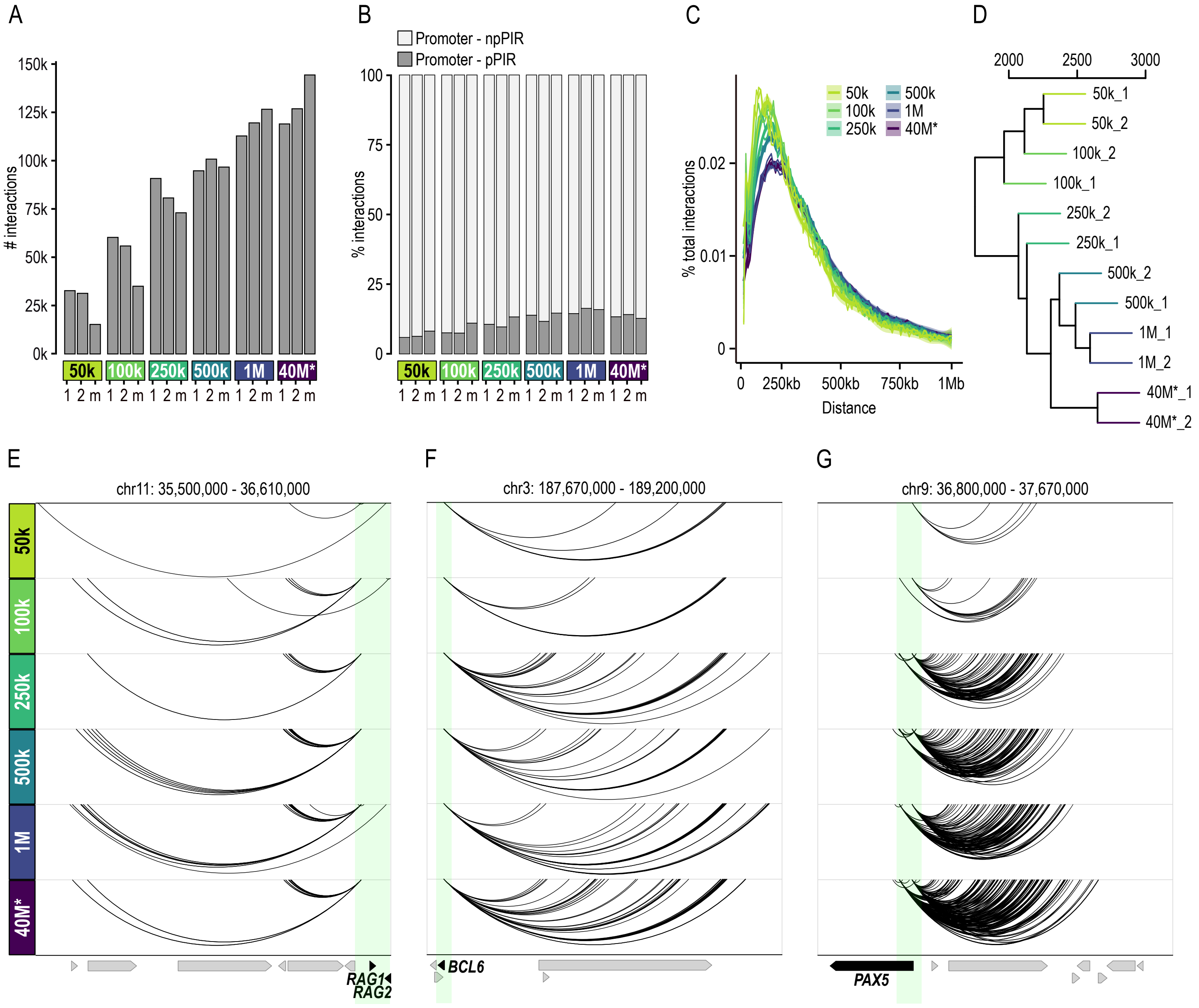

A
B
C
D
E
F
G
150k
125k
100k
75k
50k
25k
0k
interactions
50k
100k
250k
500k
1M
40M*
1 2 m
Promoter - npPIR
Promoter - pPIR
% interactions
100
75
50
25
0
% total interactions
0.02
0.01
0
250kb
500kb
750kb
1Mb
Distance
2000
2500
3000
50k_1
50k_2
100k_2
100k_1
250k_2
250k_1
500k_2
500k_1
1M_1
1M_2
40M*_1
40M*_2
chr11: 35,500,000 - 36,610,000
chr3: 187,670,000 - 189,200,000
chr9: 36,800,000 - 37,670,000
RAG1
RAG2
BCL6
PAX5

Supplementary Fig. 2 | A Total number of CHiCAGO significant interactions (score >5) of biological replicates (1 and 2) and merged samples (m) obtained by PCHi-C (*) and liCHi-C using different numbers of naive B cells as input material. 1000 (k), million (M). **B** Proportions of promoter-npPIR and promoter-pPIR of CHiCAGO significant interactions of biological replicates. **C** Distance distribution of CHiCAGO significant interactions of merged samples. **D** Dendrogram of hierarchical clustering with average linkage based on Euclidean distances of CHiCAGO significant interactions of biological replicates. *RAG1/2* (**E**) *BCL6* (**F**) and *PAX5* (**G**) promoter-centered interactions (arcs) according to liCHi-C data. Green shade depicts gene promoters, while yellow shades depict putative enhancer regions for that gene. Arrows symbolize gene placement and orientation along the genomic window.

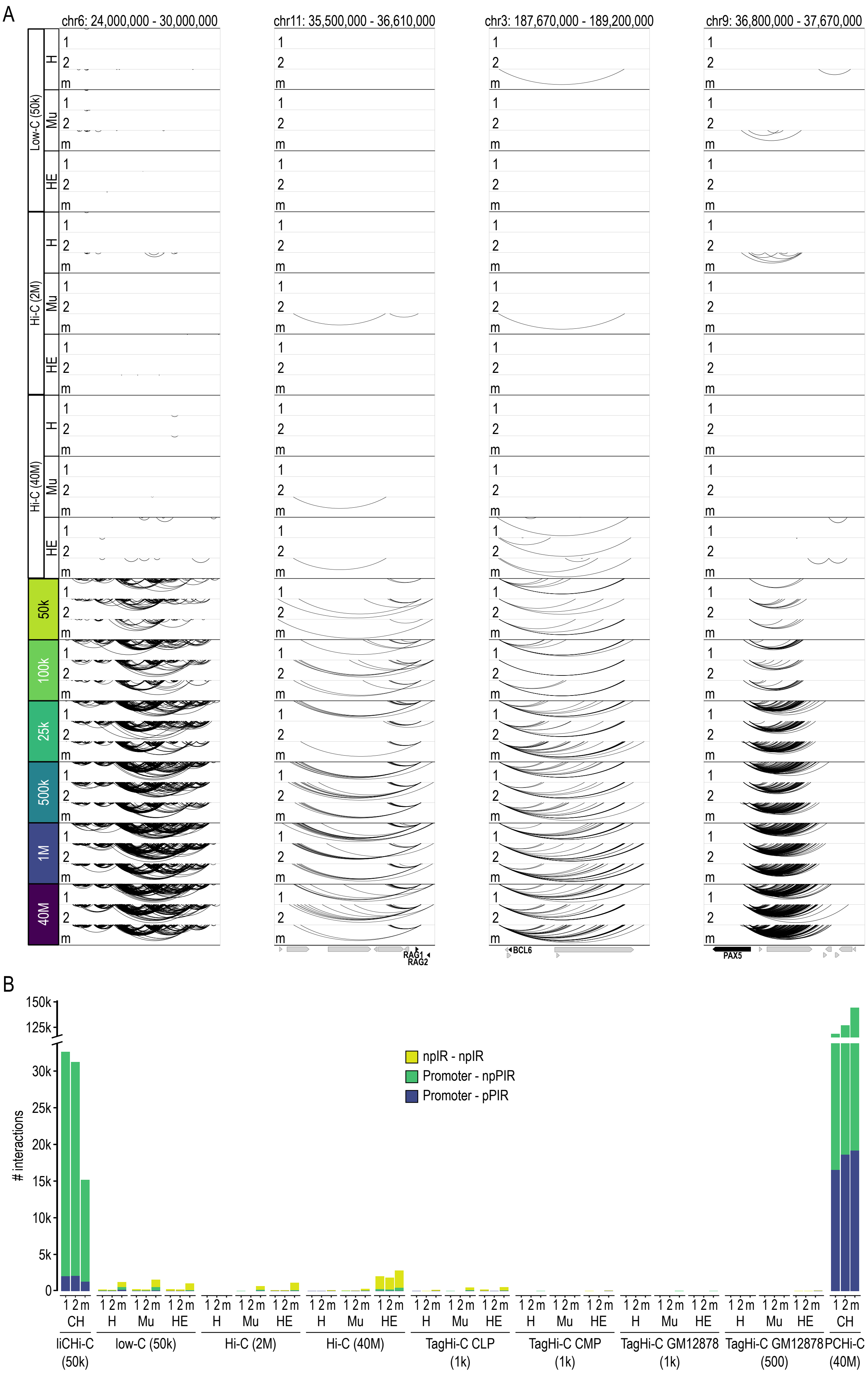
A
chr6: 24,000,000 - 30,000,000
chr11: 35,500,000 - 36,610,000
chr3: 187,670,000 - 189,200,000
chr9: 36,800,000 - 37,670,000
Low-C (50k)
Hi-C (2M)
Hi-C (40M)
H
Mu
HE
50k
100k
25k
500k
1M
40M
1
2
m
RAG1
RAG2
BCL6
PAX5
B
interactions
150k
125k
30k
25k
20k
15k
10k
5k
0
npIR - npIR
Promoter - npPIR
Promoter - pPIR
1 2 m
CH
liCHi-C (50k)
low-C (50k)
Hi-C (2M)
Hi-C (40M)
TagHi-C CLP (1k)
TagHi-C CMP (1k)
TagHi-C GM12878 (1k)
TagHi-C GM12878 (500)
PCHi-C (40M)

Supplementary Fig. 3 | A Side-by-side comparisons of significant interactions at selected loci from Fig. 1D and Supplementary Fig. 2E-G detected in human naive B cells using liCHi-C, PCHi-C. Low-C and Hi-C methods. Significant loops were called in each biological replicate (1 and 2) and at the merged level (m) using standard parameters and different loop callers. Specifically, liCHi-C and PCHi-C data was analyzed by CHiCAGO (CH) at the HindIII restriction fragment resolution. Hi-C and Low-C data was analyzed by HiCCUPS (H), Mustache (Mu) and HiCExplorer (HE) at the 5kb resolution. Cell numbers per biological replicate are indicated in each case. 1000 (k), million (M). **B** Number of significant interactions detected by different 3C-based methods and starting material. Significant loops were called in each biological replicate (1 and 2) and at the merged level (m). Loops from liCHi-C and PCHi-C were called by CHiCAGO (CH) at the HindIII restriction fragment resolution (~4096 bp). Interactions from Low-C and Hi-C were called by HiCCUPS (H), Mustache (Mu) and HiCExplorer (HE) at the 5kb resolution using standard parameters. Interactions were classified as interactions between two promoters (Promoter-pPIR; blue), a promoter and a non-promoter region (Promoter-npPIR; green) and two non-promoter interacting regions (npIR- npIR; yellow). Only loops that engage at least one gene promoter at one node (green and blue categories) have the potential to control gene transcription.

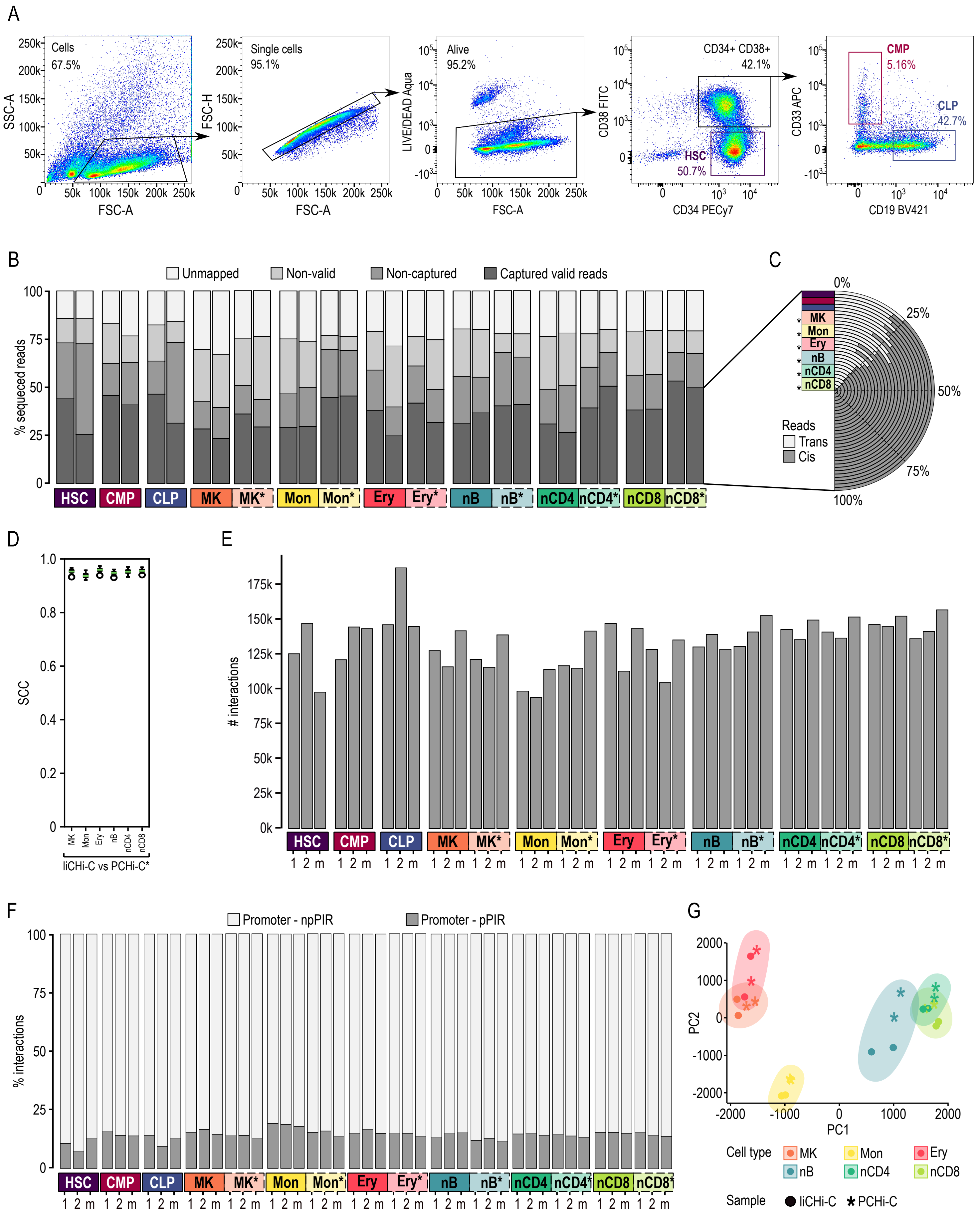
A
Cells 67.5%
SSC-A
FSC-A
Single cells 95.1%
FSC-H
Alive 95.2%
LIVE/DEAD Aqua
CD34+ CD38+ 42.1%
HSC 50.7%
CD38 FITC
CD34 PECy7
CMP 5.16%
CLP 42.7%
CD33 APC
CD19 BV421
B
Unmapped
Non-valid
Non-captured
Captured valid reads
% sequeced reads
HSC CMP CLP MK MK* Mon Mon* Ery Ery* nB nB* nCD4 nCD4* nCD8 nCD8*
C
0%
25%
50%
75%
100%
Reads
Trans
Cis
D
SCC
liCHi-C vs PCHi-C*
E
interactions
F
Promoter - npPIR
Promoter - pPIR
% interactions
G
PC1
PC2
Cell type
MK Mon Ery nB nCD4 nCD8
Sample
liCHi-C
PCHi-C

Supplementary Fig. 4 | A Schematic FACS sequential sorting strategies to isolate hematopoietic stem cell (HSC), common myeloid progenitor (CMP), common B cell lymphoid progenitor (CLP). **B** Proportions of liCHi-C reads passed through the different steps of HiCUP. Hematopoietic stem cell (HSC), common myeloid progenitor (CMP), common B-cell lymphoid progenitor (CLP), megakaryocytes (MK), monocytes (Mon), erythroblast (Ery), naive B cell (nB), naive $CD4^+$ cells (nCD4) and naive $CD8^+$ cells (nCD8). **C** Cis-trans interaction ratio of liCHi-C valid captured reads. Cis and trans mean interactions within and between chromosome respectively. **D** Boxplots of the stratum adjusted correlation coefficient (SCC) between promoter interactomes obtained by PCHi-C and liCHi-C. Medians are represented by green lines. n=2 biologically independent libraries examined over 12 independent experiments. Boxes expand the interquartile range (IQR), *i.e.,* from the first quartile (Q1) to the third quartile (Q3) values of the data, where the green lines represent the median. Whiskers correspond to the highest and the lowest points with 1.5xIQR. **E** Total number of CHiCAGO significant interactions (score > 5) called in biological replicates (1 and 2) and merged samples (m). **F** Proportions of promoter-npPIR and promoter-pPIR of significant interactions of biological replicates (1 and 2) and merged samples (m) detected by liCHi-C and PCHi-C. **G** Principal component analysis of CHiCAGO significant interactions called in each biological replicate of liCHi-C and PCHi-C.

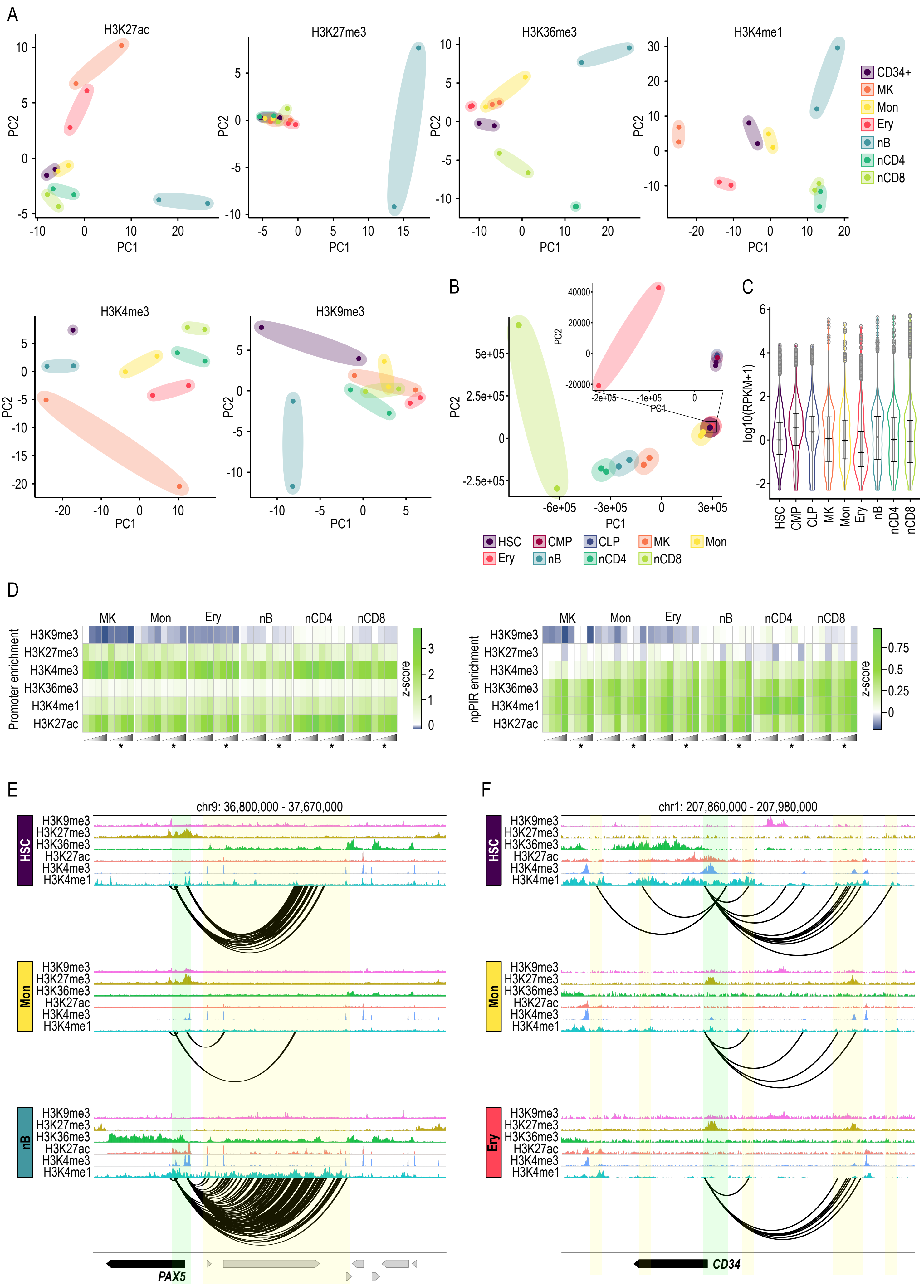
A
H3K27ac
H3K27me3
H3K36me3
H3K4me1
CD34+
MK
Mon
Ery
nB
nCD4
nCD8
H3K4me3
H3K9me3
PC1
PC2
B
C
HSC
CMP
CLP
log10(RPKM+1)
D
Promoter enrichment
npPIR enrichment
z-score
E
chr9: 36,800,000 - 37,670,000
PAX5
F
chr1: 207,860,000 - 207,980,000
CD34

Supplementary Fig. 5 | Principal component analysis plot of the significant ChIP-seq peaks from 6 different histone marks (**A**) and FPKM expression values (**B**). Zoom-in of some samples are shown for clear visualization. Hematopoietic stem cell (HSC), common myeloid progenitor (CMP), common B-cell lymphoid progenitor (CLP), megakaryocytes (MK), monocytes (Mon), erythroblast (Ery), naive B cell (nB), naive $CD4^{+}$ cells (nCD4) and naive $CD8^{+}$ cells (nCD8). **C** Violin plot representing FPKM expression values of merged RNA-seq samples, highlighting the quartiles cut-offs used to classify significant interactions by the expression of its genes. n=2 biologically independent libraries examined over 5 independent experiments. First quartile (Q1), median and third quartile (Q3) are indicated by horizontal lines. **D** Heatmap representing enrichment (expressed in terms of z-scores) of promoters (left) and non-promoter promoter-interacting regions (npPIRs) (right) for histone marks. Promoter-interacting regions (PIRs) and promoters were identified by PCHi-C using 40 million cells and classified by the quartiles level of FPKM expression of the associated gene. **E** *PAX5* promoter-centered interaction landscape (arcs) in hematopoietic stem cells (top), monocytes (middle) and naive B cells (bottom). Green shade depicts the gene promoter, while yellow shades depict putative enhancer regions for that gene in any of these cell types. **F** *CD34* promoter-centered interaction landscape (arcs) in hematopoietic stem cells (top), monocytes (middle) and erythrocytes (bottom). Green shade depicts the gene promoter, while yellow shades depict putative enhancer regions for that gene in any of these cell types.

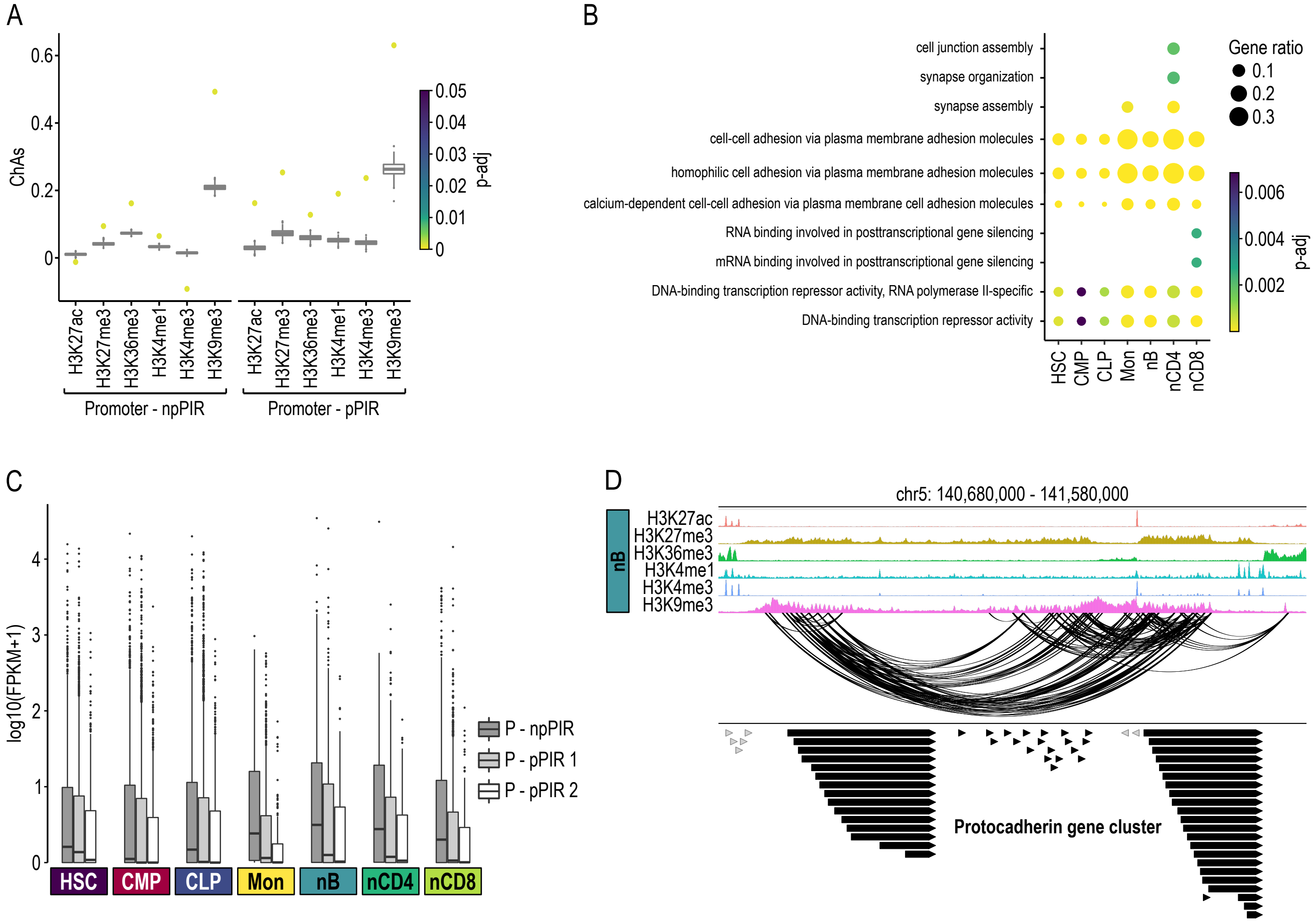
A
ChAs
0.6
0.4
0.2
0
p-adj
0.05
0.04
0.03
0.02
0.01
0
H3K27ac
H3K27me3
H3K36me3
H3K4me1
H3K4me3
H3K9me3
Promoter - npPIR
Promoter - pPIR
B
cell junction assembly
synapse organization
synapse assembly
cell-cell adhesion via plasma membrane adhesion molecules
homophilic cell adhesion via plasma membrane adhesion molecules
calcium-dependent cell-cell adhesion via plasma membrane cell adhesion molecules
RNA binding involved in posttranscriptional gene silencing
mRNA binding involved in posttranscriptional gene silencing
DNA-binding transcription repressor activity, RNA polymerase II-specific
DNA-binding transcription repressor activity
HSC
CMP
CLP
Mon
nB
nCD4
nCD8
Gene ratio
0.1
0.2
0.3
p-adj
0.006
0.004
0.002
C
log10(FPKM+1)
4
3
2
1
0
P - npPIR
P - pPIR 1
P - pPIR 2
HSC
CMP
CLP
Mon
nB
nCD4
nCD8
D
chr5: 140,680,000 - 141,580,000
nB
H3K27ac
H3K27me3
H3K36me3
H3K4me1
H3K4me3
H3K9me3
Protocadherin gene cluster

Supplementary Fig. 6 | A Boxplot displaying chromatin assortativity (ChAs) values of 1000 distance-preserved randomized networks. ChAs values of the liCHi-C promoter-npPIRs subnetwork (left) and promoter-pPIR subnetwork (right) for each histone mark in common myeloid progenitor cells are represented with colored dots. Color represents adjusted p-value. n=2 biologically independent libraries examined over 6 independent experiments. Boxes expand the interquartile range (IQR), *i.e.*, from the first quartile (Q1) to the third quartile (Q3) values of the data, where the middle lines represent the median. Whiskers correspond to the highest and the lowest points with 1.5xIQR. p values were calculated using a two-tailed permutation test. **B** Gene Ontology terms enrichment for genes involved in H3K9me3-centred interaction networks based on liCHi-C data. Hematopoietic stem cell (HSC), common myeloid progenitor (CMP), common B-cell lymphoid progenitor (CLP), megakaryocytes (MK), monocytes (Mon), erythroblast (Ery), naive B cell (nB), naive $CD4^+$ cells (nCD4) and naive $CD8^+$ cells (nCD8). Color and point size represent adjusted p-value and gene ratio respectively. p values were calculated using a hypergeometric distribution test and adjusted for multiple comparison using a False Discovery Rate (FDR) cutoff of 0.05. **C** Boxplots of log10 FPKM expression values of the genes involved in H3K9me3-mediated promoter interaction networks. Interactions with peak of H3K9me3 in the npPIRs interacting with a promoter (P-npPIR), interactions with a H3K9me3 peak in one of the two interacting promoters (P-pPIR 1), interactions with H3K9me3 peaks in both interacting promoters (P-pPIR 2). n=2 biologically independent libraries examined over 7 independent experiments. Boxes expand the interquartile range (IQR), where the middle lines represent the median. Whiskers correspond to the highest and the lowest points with 1.5xIQR. **D** H3K9me3-mediated promoter interaction network centered on the protocadherin genes cluster in naive B cells. Arrows symbolize gene placement and orientation along the genomic window.

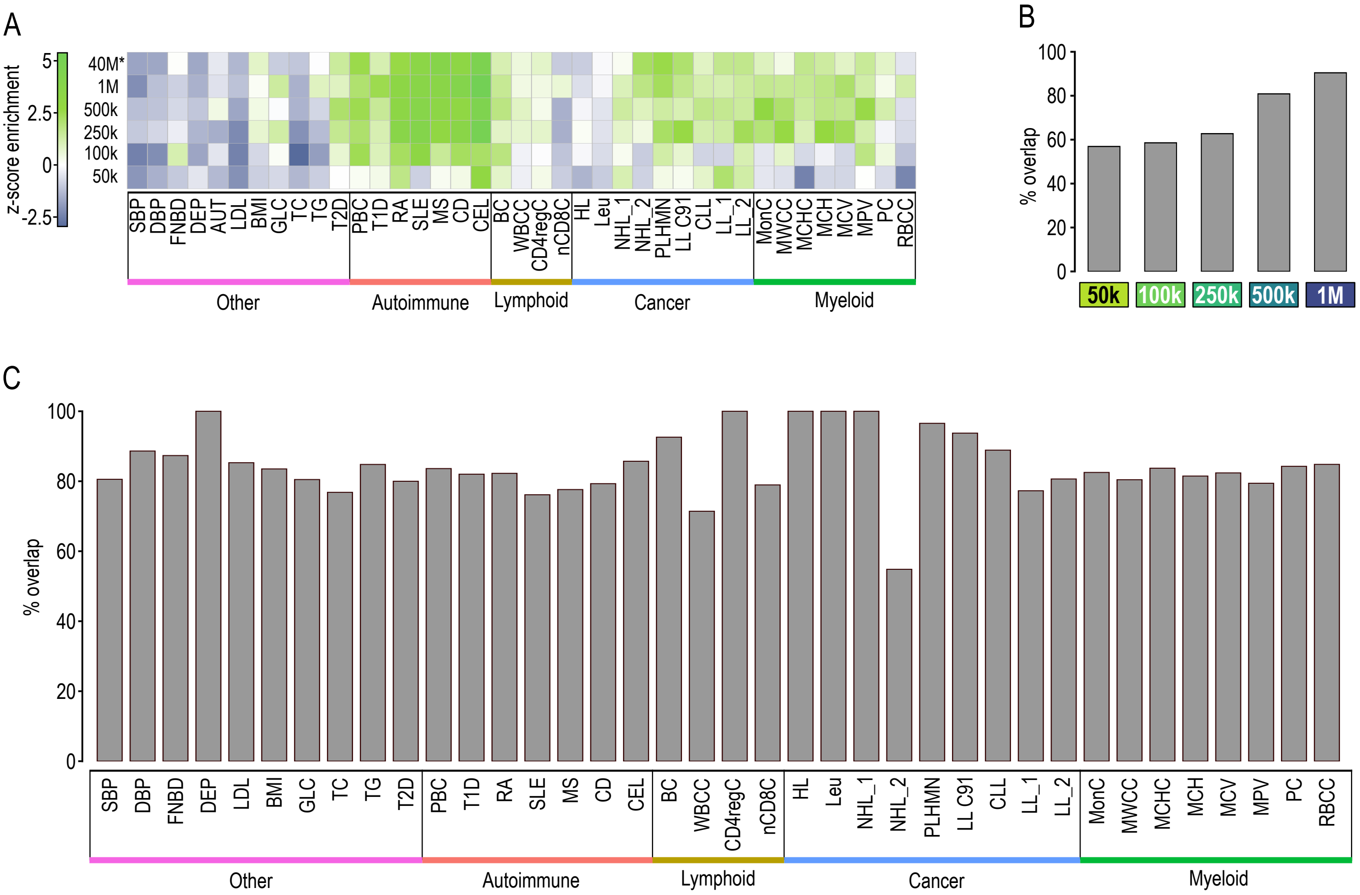
A
z-score enrichment
5
2.5
0
-2.5
40M*
1M
500k
250k
100k
50k
SBP DBP FNBD DEP AUT LDL BMI GLC TC TG T2D PBC T1D RA SLE MS CD CEL BC WBCC CD4regC nCD8C HL Leu NHL_1 NHL_2 PLHMN LL C91 CLL LL_1 LL_2 MonC MWCC MCHC MCH MCV MPV PC RBCC
Other
Autoimmune
Lymphoid
Cancer
Myeloid
B
% overlap
100
80
60
40
20
0
50k
100k
250k
500k
1M
C
% overlap
100
80
60
40
20
0
SBP DBP FNBD DEP LDL BMI GLC TC TG T2D PBC T1D RA SLE MS CD CEL BC WBCC CD4regC nCD8C HL Leu NHL_1 NHL_2 PLHMN LL C91 CLL LL_1 LL_2 MonC MWCC MCHC MCH MCV MPV PC RBCC
Other
Autoimmune
Lymphoid
Cancer
Myeloid

Supplementary Fig. 7 | A Heatmap of Blockshifter z-scores reflecting the enrichment of SNPs at promoter-interacting regions (PIRs) called by liCHi-C. For each trait, comparisons have been made between each naive B cell titration against the control endothelial precursors. Green indicates enrichment in naive B cells; blue indicates enrichment in the endothelial cell control. Traits are labeled and colored by category: autoimmune disorder (red), lymphoid trait (yellow), myeloid trait (green), blood cancer (blue) and other (purple). Systolic blood pressure (SBP), diastolic blood pressure (DBP), femoral neck bone mineral density (FNBD), depression (DEP) autism (AUT), low density lipoprotein (LDL), body mass index (BMI), glucose sensitivity (GLC), total cholesterol (TC), triglycerides (TG), type II diabetes (T2D), primary biliary cirrhosis (PBC), type I diabetes (T1D), rheumatoid arthritis (RA), systemic lupus erythematosus (SLE), multiple sclerosis (MS), Crohn's disease (CD), celiac disease (CEL), B cell absolute count (BC), white blood cell count (WBCC), CD4 regulatory T cell absolute count (CD4regC), Naive $CD8^{+}$ T cell absolute count (nCD8C), Hodgkins lymphoma (HL), leukemia (Leu), non-Hodgkins lymphoma_1 (NHL_1:), non-Hodgkins lymphoma_2 (NHL_2), primary lymphoid and hematopoietic malignant neoplasms (PLHMN), lymphoid leukemia (LLC91), chronic lymphocytic leukemia (CLL), lymphoid leukaemia_1 (LL_1), lymphoid leukaemia_2 (LL_2), monocyte count (MC), myeloid white cell count (MWCC), mean corpuscular hemoglobin concentration (MCHC), mean corpuscular hemoglobin (MCH), mean corpuscular volume (MCV), mean platelet volume (MPV), platelet count (PC), red blood cell count (RBCC). 1000 (k), million (M). **B** Percentage of overlap between the set of genes prioritized by COGS between naive B cells liCHi-C against the set of genes prioritized with PCHi-C using 40M sample for lymphoid traits. **C** Percentage of overlap between the set of genes prioritized by COGS with liCHi-C against the set of genes prioritized with PCHi-C. Unlike in Supplementary Fig. 7B, the 6 cell types and all GWAS datasets were included in the analysis.

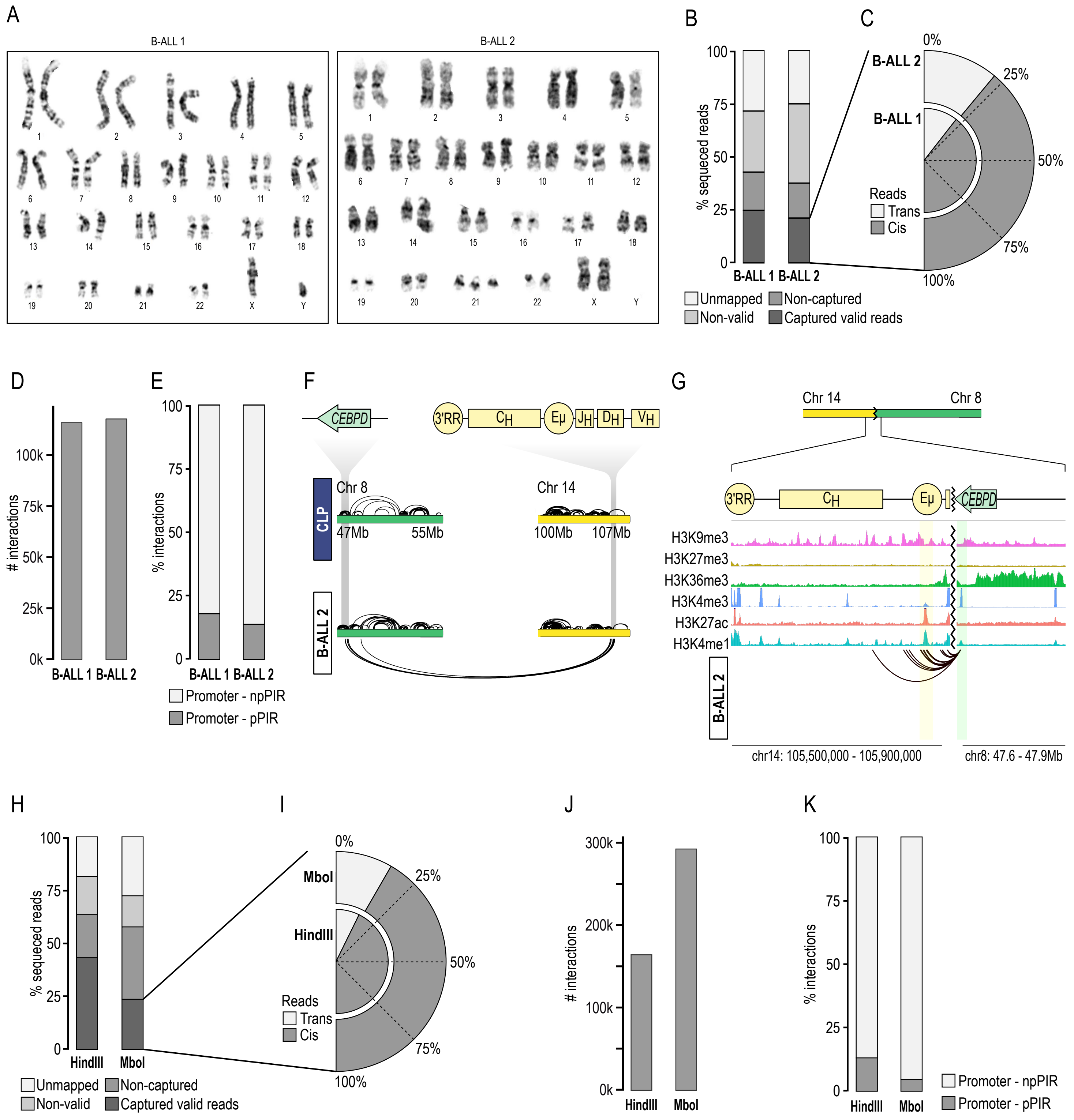

A
B-ALL 1
B-ALL 2
B
% sequeced reads
B-ALL 1 B-ALL 2
Unmapped
Non-captured
Non-valid
Captured valid reads
C
B-ALL 2
B-ALL 1
Reads
Trans
Cis
0%
25%
50%
75%
100%
D
interactions
B-ALL 1 B-ALL 2
E
% interactions
Promoter - npPIR
Promoter - pPIR
F
CEBPD
3'RR
CH
Eμ
JH
DH
VH
CLP
B-ALL 2
Chr 8
Chr 14
47Mb
55Mb
100Mb
107Mb
G
Chr 14
Chr 8
H3K9me3
H3K27me3
H3K36me3
H3K4me3
H3K27ac
H3K4me1
chr14: 105,500,000 - 105,900,000
chr8: 47.6 - 47.9Mb
H
% sequeced reads
HindIII
MboI
I
MboI
HindIII
J
interactions
K
% interactions

Supplementary Fig. 8 | A G-band karyotype of pediatric B-cell precursor acute lymphoblastic leukemia (B-ALL) 1 and B-ALL 2 samples. **B** Proportions of liCHi-C reads passed through the different steps of HiCUP. **C** Cis-trans interaction ratio of liCHi-C valid captured reads. Cis and trans mean interactions within and between chromosome respectively. **D** Total number of liCHi-C significant interactions. **E** Proportions of promoter-npPIR and promoter-pPIR of liCHi-C significant interactions. **F** Top: Schematic representation of *CEBPD* and *IgH* gene loci. Bottom: interaction landscape of chromosomes 8 and 14 in common B-cell lymphoid progenitors (CLP) and B-ALL 2 sample. Interactions within and between chromosomes are represented over and below the chromosomes respectively. **G** Reconstruction of *CEBPD* promoter interaction landscape on the derivative chromosome in B-ALL 2. Green shade depicts the *CEBPD* promoter and the yellow shadow the enhancer μ (Eμ) of the *IgH* loci. **H** Proportions of liCHi-C reads from B-ALL patient 3 passed through the different steps of HiCUP. **I** Cis-trans interaction ratio of liCHi-C valid captured reads from B-ALL patient 3 generated with HindIII and MboI restriction enzyme. Cis and trans mean interactions within and between chromosome respectively. **J** Total number of liCHi-C significant interactions from B-ALL patient 3. **K** Proportions of promoter-npPIR and promoter-pPIR of liCHi-C significant interactions detected using HindIII and MboI digestion respectively.
