# Supplementary material for: Low input capture Hi-C (liCHi-C) identifies promoter-enhancer interactions at high-resolution
Source: Nat Commun. 2023 Jan 17;14:268. doi: 10.1038/s41467-023-35911-8 (PMC9845235; doi:10.1038/s41467-023-35911-8)
Supplement: Supplementary file 3 — Description of additional Supplementary File [file 41467_2023_35911_MOESM3_ESM.pdf]

Descriptions of additional supplementary files

Supplementary Data 1. liCHiC libraries statistics

Supplementary Data 2. Benchmarking liCHi-C against with other existing 3C- based methods

Supplementary Data 3. RNA-seq & ChIP-seq datasets.

Supplementary Data 4. GWAS datasets

Supplementary Data 5. Primers, adapters, and PCR conditions
