# Supplementary material for: Low input capture Hi-C (liCHi-C) identifies promoter-enhancer interactions at high-resolution
Source: Nat Commun. 2023 Jan 17;14:268. doi: 10.1038/s41467-023-35911-8 (PMC9845235; doi:10.1038/s41467-023-35911-8)
Supplement: Supplementary file 9 — Reporting Summary [file 41467_2023_35911_MOESM9_ESM.pdf]

nature portfolio

Corresponding author(s): Biola M. Javierre

Last updated by author(s): May 25, 2022

Reporting Summary

Nature Portfolio wishes to improve the reproducibility of the work that we publish. This form provides structure for consistency and transparency in reporting. For further information on Nature Portfolio policies, see our Editorial Policies and the Editorial Policy Checklist.

Statistics

For all statistical analyses, confirm that the following items are present in the figure legend, table legend, main text, or Methods section.

n/a	Confirmed	
[ ]	[x]	The exact sample size (*n*) for each experimental group/condition, given as a discrete number and unit of measurement
[ ]	[x]	A statement on whether measurements were taken from distinct samples or whether the same sample was measured repeatedly
[ ]	[x]	The statistical test(s) used AND whether they are one- or two-sided<br>*Only common tests should be described solely by name; describe more complex techniques in the Methods section.*
[x]	[ ]	A description of all covariates tested
[ ]	[x]	A description of any assumptions or corrections, such as tests of normality and adjustment for multiple comparisons
[ ]	[x]	A full description of the statistical parameters including central tendency (e.g. means) or other basic estimates (e.g. regression coefficient) AND variation (e.g. standard deviation) or associated estimates of uncertainty (e.g. confidence intervals)
[ ]	[x]	For null hypothesis testing, the test statistic (e.g. *F*, *t*, *r*) with confidence intervals, effect sizes, degrees of freedom and *P* value noted<br>*Give P values as exact values whenever suitable.*
[x]	[ ]	For Bayesian analysis, information on the choice of priors and Markov chain Monte Carlo settings
[x]	[ ]	For hierarchical and complex designs, identification of the appropriate level for tests and full reporting of outcomes
[x]	[ ]	Estimates of effect sizes (e.g. Cohen's *d*, Pearson's *r*), indicating how they were calculated

Our web collection on statistics for biologists contains articles on many of the points above.

Software and code

Policy information about availability of computer code

Data collection	No software was used for data collection.
Data analysis	Software versions: BD FACSDiva™ Software 8.0.2; HiCUP 0.8.2; CHiCAGO 1.14.0; AutoClass 3.3.6; Trim Galore 0.6.5; bowtie2 2.3.2; macs2 2.2.7.1; deepTools 3.2.1; RegioneReloaded 1.0.0; ChAseR 0.0.0.9 (https://bitbucket.org/eraineri/chaser); clusterProfiler 4.2.2; ReactomePA 1.38.0; bam2pairs 0.3.7 (https://github.com/4dn-dcic/pairix); cooler 0.8.11; HiCExplorer 3.7.2; WashU Epigenome Browser Legacy 46.2; karyoploteR 1.22.0; UCSC liftOver tool (Nov 2021 release); qqman 0.1.8; VEP 104; PLIER 0.21 (https://github.com/deLaatLab/PLIER); Control-FREEC 11.5. Scripts to compute specificity score have been adapted from https://github.com/Steven-M-Hill/PCHiC-specificity-score-analysis; and implementations of PMI, Blockshifter and COGS algorithm from https://github.com/ollyburren/CHIGP. All custom code and the required software versions are publicly available in GitHub at https://github.com/JavierreLab/liCHiC.

For manuscripts utilizing custom algorithms or software that are central to the research but not yet described in published literature, software must be made available to editors and reviewers. We strongly encourage code deposition in a community repository (e.g. GitHub). See the Nature Portfolio guidelines for submitting code & software for further information.

Data

Policy information about availability of data

All manuscripts must include a data availability statement. This statement should provide the following information, where applicable:

- Accession codes, unique identifiers, or web links for publicly available datasets
- A description of any restrictions on data availability
- For clinical datasets or third party data, please ensure that the statement adheres to our policy

Due to the potentially identifiable nature of the data, raw liCHi-C datasets have been deposited to EGA (https://www.ebi.ac.uk/ega) and are available upon request under the accession number EGAS00001006305. Reference genomes were obtained from Ensembl: GRCh38.p13 (release 104) and GRCm39 (release 106). The 1000 Genomes variants data was downloaded from the International Genome Sample Resource (data collection: 1000 Genomes on GRCh38), and the GRCh38 genetic map was obtained from http://csg.sph.umich.edu/locuszoom/download/recomb-hg38.tar.gz. PCHi-C data analysed in the study is available at EGA (accession number: EGAS00001001911). Data used for the benchmarking of C-based methods was publicly available under the following accession codes: E-MTAB-5875 at ArrayExpress for Low-C data; EGAS00001004763 and EGAS00001001911 at EGA for Hi-C data; GSE161082 and GSE152918 at Gene Expression Omnibus (GEO) for TagHi-C data. Futher details are shown in Supplementary Data 2. Data of publicly available omics including ChIP-seq and RNA-seq was obtained from BLUEPRINT (http://dcc.blueprint-epigenome.eu) and ROADMAP (http://www.roadmapepigenomics.org); and GWAS Summary Statistics were downloaded from the NHGRI-EBI GWAS Catalog (https://www.ebi.ac.uk/gwas) and from the UK Biobank - Neale Lab (http://www.nealelab.is/uk-biobank). Accession codes for ChIP-seq and RNA-seq are presented in Supplementary Data 3; for GWAS Summary Statistics, see Supplementary Data 4.

Human research participants

Policy information about studies involving human research participants and Sex and Gender in Research.

Reporting on sex and gender	Our study and findings do not take into consideration sex or gender, nor our findings apply to only one sex or gender due to the nature of the samples and the analysis.
Population characteristics	Haematopoietic progenitors were isolated from fetal donations ranging from 15-22 post-conception weeks fetal donations. No other population characteristics were taken into account in the study.
Recruitment	No participants / volunteers were recruited for our research
Ethics oversight	The study was conducted according to the guidelines of the Declaration of Helsinki and approved by the Institutional Review Board of the Clinical Research Ethics Committee of University Hospital Germans Trias i Pujol REF.CEI: PI-18-205

Note that full information on the approval of the study protocol must also be provided in the manuscript.

Field-specific reporting

Please select the one below that is the best fit for your research. If you are not sure, read the appropriate sections before making your selection.

[x] Life sciences [ ] Behavioural & social sciences [ ] Ecological, evolutionary & environmental sciences

For a reference copy of the document with all sections, see nature.com/documents/nr-reporting-summary-flat.pdf

Life sciences study design

All studies must disclose on these points even when the disclosure is negative.

Sample size	As sample size we used 2 biological replicates for each condition. Sample size was determined based on similar capture Hi-C experiments in previous studies (1,2), allowing a reduction of heterogeneity bias while keeping the experiments at a non-prohibitive cost.<br><br>1. Thiecke MJ et al. Cohesin-Dependent and -Independent Mechanisms Mediate Chromosomal Contacts between Promoters and Enhancers. Cell Rep. 2020 Jul 21;32(3):107929.<br>2. Zhang C et al. tagHi-C Reveals 3D Chromatin Architecture Dynamics during Mouse Hematopoiesis. Cell Rep. 2020 Sep 29;32(13):108206.
Data exclusions	No data was excluded from the analysis
Replication	For the nB titration and the HSC, CMP and CLP, 3 relicates were performed but only 2 used since one failed in the library preparation.<br>For the differentitated cell types, 2 replicates were performed and used since they worked at first try.<br>For the B-ALL clinical samples, only 1 replicate was performed due to patient sample availability.<br><br>Experimental reproducibility between liCHi-C with different numbers of input cells and with PCHi-C was measured using reproducibility-score as described in Yang et al., Genome Res 2017.

Randomization	Randomization is not relevant as we did not allocate datasets into experimental groups
Blinding	Blinding is not necessary as we did not allocate datasets into experimental groups

Reporting for specific materials, systems and methods

We require information from authors about some types of materials, experimental systems and methods used in many studies. Here, indicate whether each material, system or method listed is relevant to your study. If you are not sure if a list item applies to your research, read the appropriate section before selecting a response.

Materials & experimental systems

n/a	Involved in the study
☐	☒ Antibodies
☒	☐ Eukaryotic cell lines
☒	☐ Palaeontology and archaeology
☒	☐ Animals and other organisms
☒	☐ Clinical data
☒	☐ Dual use research of concern

Methods

n/a	Involved in the study
☒	☐ ChIP-seq
☐	☒ Flow cytometry
☒	☐ MRI-based neuroimaging

Antibodies

Antibodies used	CD34 PECy7 (BD Biosciences, cat. #348811, clone 8G12, lot 1333095)<br>CD38 FITC (BD Biosciences, cat. #555459, clone HIT2, lot 63288698)<br>CD19 BV421 (BD Biosciences, cat. #562440, clone HIB19, lot 1063075)<br>CD33 APC (BD Biosciences, cat. #551378, clone WM53, lot 8101518)
Validation	All primary antibodies were shown to react to their target protein on the manufacturer's website and complies to all BD release criteria according to each certificate of analysis corresponding to each lot

Flow Cytometry

Plots

Confirm that:

☒ The axis labels state the marker and fluorochrome used (e.g. CD4-FITC).

☒ The axis scales are clearly visible. Include numbers along axes only for bottom left plot of group (a 'group' is an analysis of identical markers).

☒ All plots are contour plots with outliers or pseudocolor plots.

☒ A numerical value for number of cells or percentage (with statistics) is provided.

Methodology

Sample preparation	Haematopoietic progenitors were isolated from fetal liver and fetal bone marrow from donations. Liver was mechanically disgregated and mononuclear cells were separated by Ficoll gradient. Bone marrow was mechanically flushed from long bones. Cells were pooled and stained with CD34 beads (Miltenyi #130-046-703) for CD34 positive selection. Afterwards, cells were labelled for FACS sorting using the described antibodies.<br><br>Naïve B, naïve CD4, naïve CD8 and monocytes were extracted by immunomagnetic selection from buffy coat donations (as described in Javierre et al., Cell 2016).<br>Erythroblasts and megakaryocytes were differentiated in vitro from CD34+ cells (as described in Javierre et al., Cell 2016).
Instrument	FACS was performed using a BD FACSAria Fusion.
Software	FACS Diva Software (BD) 8.0.2
Cell population abundance	HSC (CD34+ CD38-): 70.6% of parent population alive cells<br>CMP (CD34+ CD38+ CD33+ CD19-): 7.6% of parent population CD34+ CD38+ cells<br>CLP (CD34+ CD38+ CD33- CD19+): 66.2% of parent population CD34+ CD38+ cells<br><br>Cell population percentages can highly vary between isolations due to differences in organ availability (bone marrow or liver) and post-conception week of donations.
Gating strategy	Cells were separated from debris by FSC-A and SSC-A. Then singlets were selected by FSC-A and FSC-H. Alive single cells were

selected using LIVE/DEAD viability marker. HSC (CD34+ CD38-) cells are selected by plotting CD34 and CD38. The remaining CD34+ CD38+ population is separated into CMP (CD34+ CD38+ CD33+ CD19-) and CLP (CD34+ CD38+ CD33- CD19+) by plotting CD33 and CD19.

☒ Tick this box to confirm that a figure exemplifying the gating strategy is provided in the Supplementary Information.
